# Global impact of *KRAS* mutation patterns in FOLFOX treated metastatic colorectal cancer

**DOI:** 10.3389/fgene.2015.00116

**Published:** 2015-03-30

**Authors:** David M. Zocche, Carolina Ramirez, Fernando M. Fontao, Lucas D. Costa, María A. Redal

**Affiliations:** ^1^Molecular and Cellular Biology Department, Instituto Universitario del Hospital Italiano de Buenos Aires – Hospital Italiano de Buenos Aires, Buenos AiresArgentina; ^2^Instituto de Ciencias Básicas y Medicina Experimental, Instituto Universitario del Hospital Italiano de Buenos Aires – Hospital Italiano de Buenos Aires, Buenos AiresArgentina; ^3^Laboratory for Biological and Artificial Learning, Instituto de Ciencias Básicas y Medicina Experimental, Hospital Italiano de Buenos Aires, Buenos AiresArgentina

**Keywords:** KRAS mutation, colorectal cancer, prognosis, molecular biomarkers, clinical outcome, FOLFOX

## Abstract

**Background:** Colorectal cancer (CRC) is one of the most frequent events in oncology. Advances in molecular understanding of the processes of carcinogenesis have shed light on the fundamental mechanisms of tumorigenesis. Currently, knowledge of the molecular basis of its pathogenesis is being used to improve patient care and devise more rational therapeutics. Still, the role played by the mutation patterns of mutated genes in the clinical outcomes that patients on pharmacological treatment receive remains unclear. In this study, we propose to analyze the different clinical outcomes and disease prognosis of patients with stage IV CRC treated with FOLFOX chemotherapy (fluorouracil, leucovorin, oxaliplatin) based on different *Kirsten ras* (*KRAS*) mutation patterns.

**Methods:** In this cohort study, 148 patients diagnosed with stage IV CRC and treated with FOLFOX were studied between 2008 and 2013. Mutational status of *KRAS* was determined. Progression-free survival (PFS) and overall survival (OS) were measured, and all deaths were verified. Survival analysis was performed using Kaplan–Meier analysis, comparison among groups was analyzed using the log-rank test, and multivariate analysis was conducted using Cox proportional-hazards regression.

**Results:** Among a total of 148 patients, 48 (32%) had mutated *KRAS*, 77% at codon 12 and 23% at codon 13. The PFS was significantly worse in the mutant *KRAS* patients in comparison to wild type *KRAS* patients (*p* < 0.05). The OS did not show significant differences between the two groups. Multivariate analysis showed *KRAS* mutation as an independent negative prognostic factor for PFS. Among the various subtypes of *KRAS* mutation, G12D was significantly associated with a poor prognosis in PFS (*p* = 0.02).

**Conclusion:** In our population, the *KRAS* mutation had an adverse impact on the prognosis for stage IV CRC patients treated with the FOLFOX regimen.

## Introduction

Colorectal cancer (CRC) is one of the most frequent causes of cancer death in industrialized countries, and ranks third in prevalence in the United States (Siegel et al., 2013). Its occurrence is increasing in many developed and developing countries; also in China, colon cancer represents a very important cause of morbidity and mortality (Yang et al., 2004). Even so, mortality rates have declined as a result of improved treatment and efficient screening and surveillance.

The development of CRC is a multi-step process characterized by the accumulation of genetic alterations. Currently, the molecular basis of the disease’s pathogenesis is an active area of research, both in order to improve patient care, and to develop more rational therapeutics (Kim and Kim, 2014). In this context, generating prognostic factors is a major goal in oncology (Sridharan et al., 2014; Phipps et al., 2015).

Clinicians have reached a consensus (Benson et al., 2013) that treatment of CRC should be a comprehensive project that consists of surgery, adjuvant chemotherapy, and certain targeted therapies. Currently, 5-FU based chemotherapy has been recognized as the first line regimen and is utilized for adjuvant and/or neoadjuvant treatment of CRC patients. The recent incorporation of molecularly targeted drugs (Benson et al., 2013), such as anti-EGFR monoclonal antibodies, into the traditional 5-FU-based chemotherapeutic regimen (FOLFOX and FOLFIRI) improves efficacy and is now a pivotal component in the treatment of metastatic colorectal cancer (mCRC; Grothey and Allegra, 2012). Moreover, the tumor’s mutation status, especially in the *Kirsten ras* (*KRAS*) gene, is a predictive marker for response of anti-EGFR antibody therapies in patients with mCRC. So, mutation pattern G13D is associated to sensitivity to targeted therapy with anti-EGFR and the other mutations patterns are associated with no response of this regimen (Bokemeyer et al., 2012).

The *KRAS* is member of the *ras* gene family (H-, K-, and N-ras), which encodes highly similar membrane-localized G proteins with molecular weights of 21 kDa (Amado et al., 2008). All three different known proteins are capable of binding and hydrolyzing GTP and participate in a signal transmission pathway from the cytoplasm to the nucleus (Christos Karapetis et al., 2008). Members of the *ras* gene family have been recognized as key targets in tumorigenesis due to their participation in controlling multiple pathways affecting cell growth, differentiation, and apoptosis by interacting with a series of coordinators and effectors (Barbacid, 1987), as an essential component of the EGFR signaling cascade.

In particular, *KRAS* is involved in the pathogenesis of many different malignant tumors, including lung cancer, pancreatic cancer, and colon cancer (Macara et al., 1996; Cárdenas-Ramos et al., 2014).

*Kirsten ras* can acquire activating mutations in exon 2, codons 12 and 13 (Rodenhuis et al., 1987). The prevalence of *KRAS* mutations varies greatly amongst different human tumors. Previous studies support that the frequency of mutation is around 30–40% in CRC. These results are similar across different ethnic groups (Sameer et al., 2009; Cárdenas-Ramos et al., 2014; Elsamany et al., 2014).

Identifying the status of *KRAS* in each patient is important in order to determine the best therapy: patients with the wild type (WT) could receive monoclonal antibodies against EGFR (Schubbert et al., 2007), while *KRAS* mutated patients have been associated with no-response to targeted therapies and poor prognosis in different studies (De Roock et al., 2011; Dattatreya, 2013; Douillard et al., 2013).

The objective of this study is to assess the clinical outcomes in patients with stage IV CRC treated with FOLFOX in addition to evaluating the ages of the patients, *KRAS* mutation patterns and the primary tumor location.

## Materials and Methods

### Patient’s Characteristics

In this study, we designed an observational retrospective cohort with 450 CRC patients who were diagnosed with stages II, III, and IV. The time period of evaluation was 2008–2014. All patients were treated with surgery and received adjuvant FOLFOX chemotherapy based on adding or not adding a targeted therapy, depending on the mutation status of *KRAS*, availability and/or clinical status of the patient. The exclusion criteria were the following: previous chemotherapy for CRC, previous radiotherapy for CRC and history of other malignancy within 5 years.

From the initial population of 450, we selected 149 patients with stage IV disease, treated with FOLFOX-4 or modified FOLFOX-6 regimen as a first-line, medical history data available and born in Argentina. Adjuvant chemotherapy was planned for a total of 12 cycles. Patients were assessed every week during chemotherapy treatment and then at least every 6 months during the disease free period. The post-chemotherapy period assessment included medical history, physical examination, measurement of carcinoembryonic antigen level, and evaluation with computed tomography.

The information was recovered from the electronic medical records of the Hospital Italiano de Buenos Aires. The rollection of patient information was anonymous. The date of death of all patients was verified.

The research protocol was reviewed and approved by the Hospital Italiano de Buenos Aires’s institutional Ethical Committee.

### Assay to Detect Mutant *KRAS*

#### DNA Extraction

The DNA samples were obtained from macroscopically dissected formalin-fixed paraffin-embedded (FFPE) specimens cut into 10 μm thick sections. The slides from FFPE sample were deparaffinized in xylene, washed in ethanol, and rehydrated. Any tissue surrounding the tumor was carefully pared away using scalpel under microscopic observation. The purpose of paring was to ensure that tumor cells comprised over 70% of remaining specimen. After suspension in 400 μl of 100 mM Tris-EDTA buffer and proteinase K, the specimens were incubated for 3 days at 60°C. At the end of incubation, the genomic DNA was extracted using commercial kits (DNA Blood Mini Kit, QIAGEN) following the manufacturer’s protocol.

#### PCR Amplification and Sequencing

Sequences of the *KRAS* oncogene in exon 2 were amplified using the primers forward 5′ GTGTGACATGTTCTAATATAGTCA 3′ and reverse 5′ GAATGGTCCTGCACCAGTAA 3′. The primers were designed by “Primer 3” software. The PCR mixture (50 ul) contained 0.2–0.5 μg of DNA, 2 or 1.5 mm MgCl2, 10X concentrated PCR-buffer (QIAGEN), 200 μm of deoxyribonucleoside triphosphates (dATP, dCTP, dGTP, dTTP), 200 nm of each primer, and 1.25 U of HotStar Taq DNA Polymerase (QIAGEN). Amplification was achieved on a Veriti thermocycler (Applied Biosystem). After HotStarTaq DNA-polymerase activation at 95°C for 15 min, templates were denatured at 94°C for 2 min. This initial step was followed by 30 cycles of PCR, each comprising 1 min of denaturation at 94°C, 1 min of annealing at 58°C, and 1 min of extension at 72°C. In the last cycle, the extension step was prolonged for 10 min. PCR products were submitted to electrophoresis on 3% agarose gels in Tris-acetate-EDTA buffer and stained with ethidium bromide. In all cases, direct sequencing was performed using an Applied Biosystem 3730XL sequencer (PE Applied Biosystems) according to the manufacturer’s instructions on PCR products purified using a QIAGEN gel extraction kit. We performed a second independ reaction of PCR and sequencing in order to confirm the positive results. In all the cases both sense and antisense strands were sequenced.

### Statistical Analysis

The statistical evaluation of data was performed using commercially available SPSS Statistics 17.0 software. Relative frequencies and 95% confidence intervals were calculated for categorical data. The Pearson chi-square test was used to examine whether there was a relationship between *KRAS* status and patient characteristics. Continuous data was expressed as mean ± standard deviation (SD). Survival analysis was performed using Kaplan–Meier analysis, comparison among groups was analyzed using the log-rank test, and multivariate analysis was conducted using Cox proportional-hazards regression. *P*-values < 0.05 were considered to be statistically significant.

## Results

### Patient Characteristics

A total of 148 patients with mCRC were analyzed and their baseline characteristics are summarized in **Table 1**. The primary tumor location was right colon in 26%, and left colon in 74% of patients. According to the inclusion criteria, all patients received 12 cycles of chemotherapy with the FOLFOX regimen. Forty-eight patients (32%) of 148 had the *KRAS* mutations in either codon 12 or 13. The distribution of mutations was 77% in codon 12 and 23% in codon 13. Only 36% of patients in the total population had been treated with anti-EGFR therapy.

**Table 1:** Patient characteristics.

Variable	Relative frequency (95% CI)
Sex (*n* = 148)	
* Female*	66 (58–73)
* Male*	34 (27–41)
Age (*n* = 148)	
*<65 years*	57 (49–65)
*≥65 years*	43 (35–51)
Tumor location (*n* = 148)	
* Right*	26 (19–33)
* Left*	74 (67–81)
Anti-EGFR therapy (*n* = 148)	
* No*	64 (56–72)
* Yes*	36 (28–44)
KRAS (*n* = 48)	
* WT*	68 (61–75)
* MT*	32 (25–40)
Codon (*n* = 48)	
* 12*	77 (66–88)
* 13*	23 (11–35)

CI, confidence interval; WT, wild type; MT, mutated.

We observed that in our population the treatment schedule had not been met in all patients with WT *KRAS*. Approximately half of the patients (48%) with WT *KRAS* had not been treated with anti-EGFR therapy. This situation occurs because of restrictions on imports of medicines imposed by the government. In our country the anti-EGFR drugs do not occur, and patients often are harmed due to withholding at customs. Unfortunately, this deprives the best medical care available. This data is indicated in the tables.

### Prognostic Implications of *KRAS* Mutation

**FIGURE 1:**
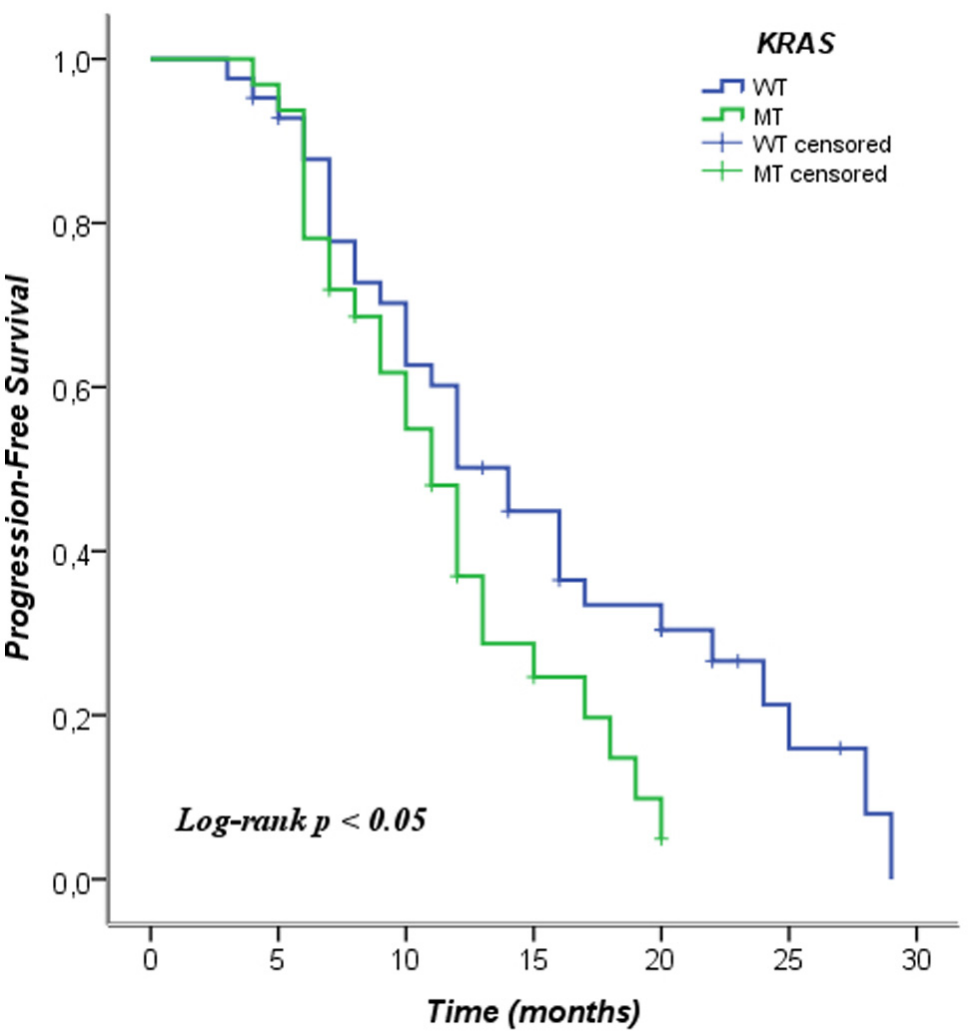
**Kaplan–Meier curve of progression-free survival according to mutation status of *KRAS*. WT, wild type; MT, mutated**.

**FIGURE 2:**
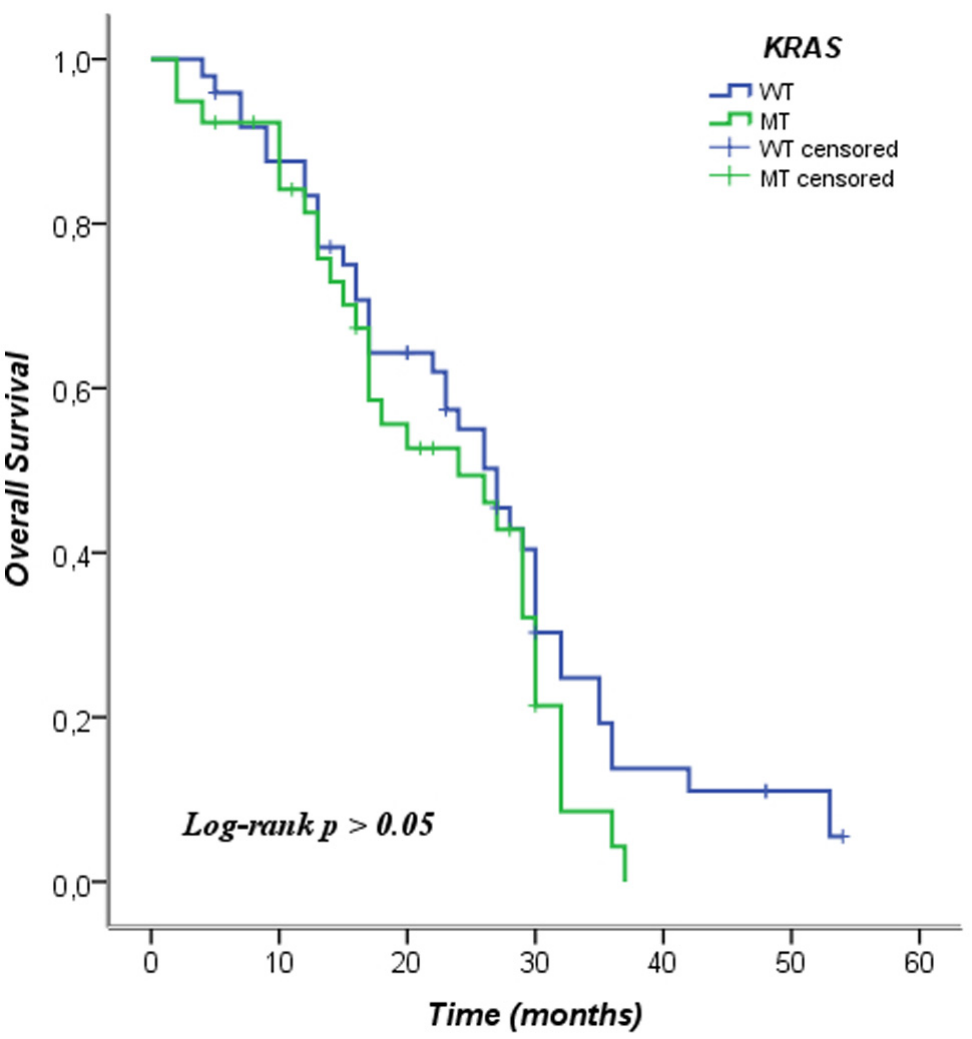
**Kaplan–Meier curve of overall survival according to mutation status of *KRAS*. WT, wild type; MT, mutated**.

We analyzed the *KRAS* mutations and characteristics of patients in the Pearson chi-square test in order to examine whether there was a relationship between them. In this analysis no relationship among the characteristics of the patients and the mutational status of *KRAS* was observed. We included patients in this analysis who were not treated with anti-EGFR therapy, even *KRAS* WT. The data is shown in **Table 2**. In **Table 3** the mean and median survival data on the patients are described. The survival analysis with the Kaplan–Meier model showed that PFS was significantly worse in mutated *KRAS* patients compared to those with WT *KRAS* w (log-rank *p* < 0.05). The Kaplan–Meier curve is shown in **Figure 1**. There was no difference in OS between patients with a mutation in *KRAS* and WT patients (**Figure 2**). In our study, multivariate analysis using the Cox proportional-hazard model showed no statistical significance for independent variables; however, the KRAS mutation status reflected a borderline *p*-value [*p* = 0.05, associated to a HR = 1.72 and a 95%CI = (0.99–2.99)] that allows to presume the influence of this variable for explaining differences in PFS (**Table 4**). None of the survival analyses included patients who had been treated with anti-EGFR therapy.

**Table 2:** Univariate analysis.

Variable	KRAS status		*p*-value
	WT	MT	
Sex (*n* = 99)			
* Female*	16	21	NS
* Male*	36	26	
Age (*n* = 99)			
*<65 years*	30	29	NS
*≥65 years*	22	18	
Tumor location (*n* = 99)			
* Right*	15	10	NS
* Left*	37	37	
Event (*n* = 88)			
* No*	10	8	NS
* Yes*	39	31	

Contingency tables and *X*^2^ test between KRAS status and patient characteristics. WT, wild type; MT, mutated, *p-value*, probability value from *X*^2^ test; NS, not significant.

**Table 3:** Median and mean survival time.

Survival	KRAS status	Median [months]	Mean [months]
Progression-Free	WT	14	15.3
Survival (PFS)	MT	11	11.6

Overall survival (OS)	WT	27	26.1
	MT	24	22.0

WT, wild type; MT, mutated.

**Table 4:** Multivariate analysis.

Variable	HR (95% CI)	*p*-value
*KRAS*	1.72 (0.99–2.99)	0.05
*Sex*	1.23 (0.71–2.16)	0.46
*Age*	0.83 (0.47–1.47)	0.51
*Tumor location*	0.85 (0.46–1.58)	0.61

Cox regression model of PFS. HR, hazard ratio; CI, confidence interval; reference categories: wild type, female, <65 years, right side.

**FIGURE 3:**
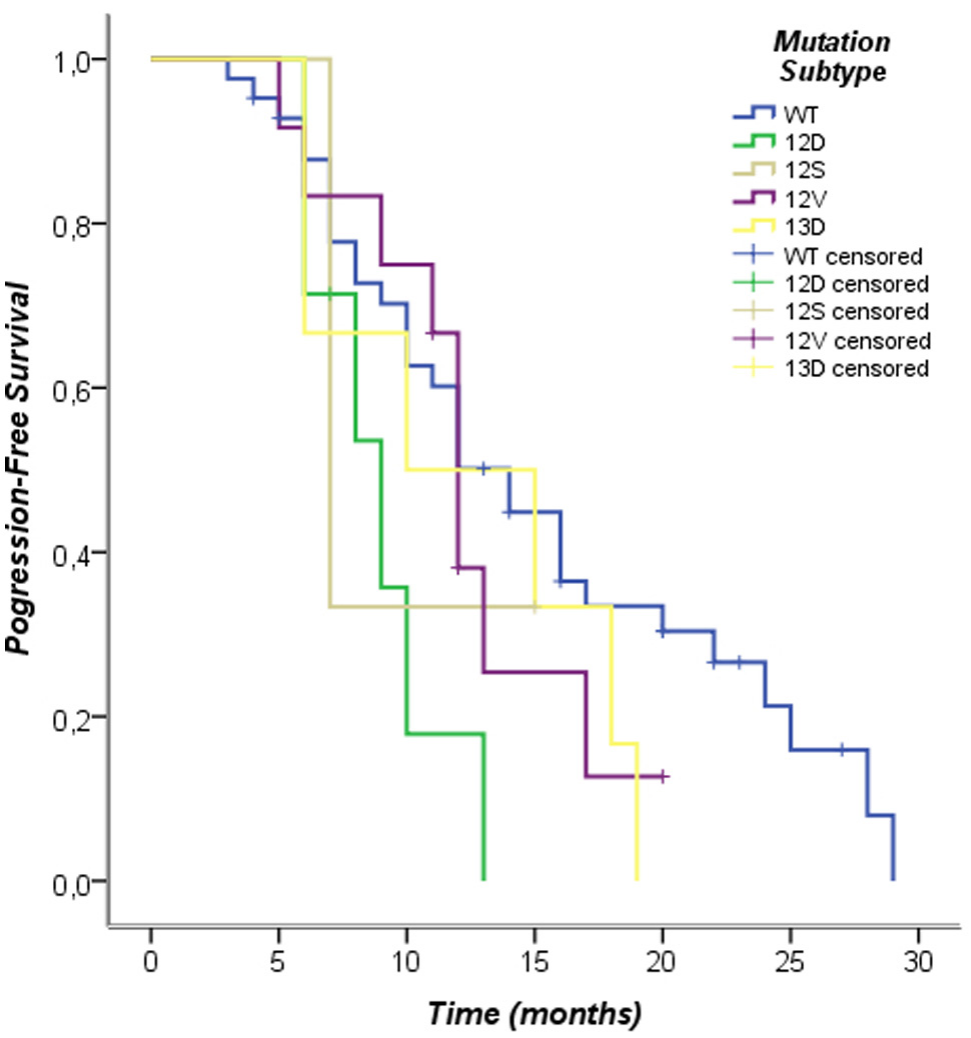
**Kaplan–Meier curves of progression-free survival according to *KRAS* mutation patterns**.

**FIGURE 4:**
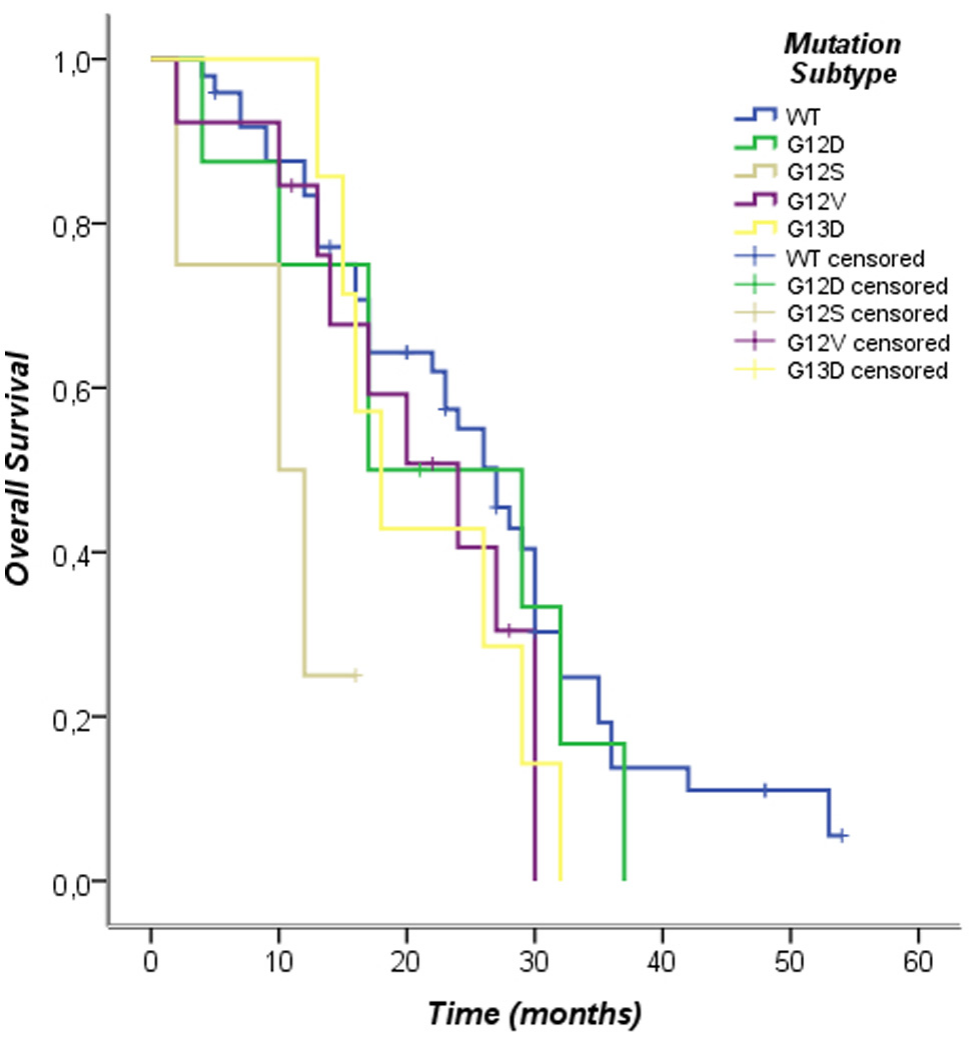
**Kaplan–Meier curves of overall survival according to *KRAS* mutation pattern**.

The Kaplan–Meier survival analysis of individual subtypes of KRAS mutation performed in this study showed the following results: the G12D subtype was associated with poor prognosis in PFS (log-rank *p* = 0.02), other subtypes mutations were not associated with differences in progression-free survival (PFS; **Figure 3**; **Table 5**). Furthermore, when analyzing the OS of individual subtypes of KRAS mutation, the G12S subtype was associated with poor prognosis (log-rank *p* = 0.01), while no significant results were shown in overall survival (OS) for other subtypes in our population The exclusion criteria were subtypes of KRAS mutations present in fewer than three cases (**Figure 4**; **Table 6**).

**Table 5:** Kaplan–Meier analysis of progression-free survival.

Subtype	*n*	*p*-value
WT	42	–
*G12D*	7	**0.02**
*G12S*	3	NS
*G12V*	12	NS
*G13D*	6	NS

Comparison according to KRAS subtype. WT, wild type; reference category, WT; *p-*value, probability value from log-rank test; NS, not significant. Statistically significant results shown in bold font.

**Table 6:** Kaplan–Meier analysis of global survival.

Subtype	*n*	*P*-value
WT	49	–
*G12D*	8	NS
*G12S*	4	**0.01**
*G12V*	13	NS
*G13D*	6	NS

Comparison according to KRAS subtype. WT, wild type; reference category, WT; *P-value*, probability value from log-rank test; NS, not significant. Statistically significant results shown in bold font.

## Discussion

Many studies indicate that *KRAS* and other mutated molecules may be important prognostic factors related to disease free-survival (Douillard et al., 2013) and OS in CRC (Samowitz et al., 2000). Computational Biology studies that analyzed the kinetics and biophysics of mutated molecules of *KRAS* had shown results regarding the instability of G12D product, as well as tridimensional configuration similar to the WT product of the G13D mutation (Chen et al., 2014).

The adverse prognosis of *KRAS* mutation on recurrence was observed in the Quick and Simple and Reliable (QUASAR) trial, which evaluated 5-FU-based adjuvant treatment (Hutchins et al., 2011). Different prognostic effects of different patterns of *KRAS* mutation have been investigated in previous studies. Samowitz et al. (2000) found the *KRAS* G13D mutation was associated with a higher risk of death in a population-based cohort. Moreover, the characterization of *KRAS* mutational status in the chemotherapy-alone subgroup of patients in the CRYSTAL and OPUS trials showed that the G13D mutation is associated with worse survival (Tejpar et al., 2012). Only the G13D mutation is sensitive to anti-EGFR monoclonal antibodies, whereas the other mutation subtypes were associated with no response (De Roock et al., 2010). In addition, another group reached the conclusion that patients can benefit from treatments with standard chemotherapy based on oxaliplatin or irinotecan when they evaluated the role of *KRAS* altogether with other mutations such as BRAF, PK3CA, NRAS, and AKT. In our population this association was not observed. Possibly these results present confounders like including patients treated with monoclonal antibodies and anti-EGFR therapies in the analysis of survival, which clearly modify survival (Soeda et al., 2014).

On the other hand, other researchers have not found a positive association between *KRAS* and the prognosis of patients (Bruera et al., 2014). In fact, the information is discordant between different studies, this is observed in a comprehensive meta-analysis (Ren et al., 2012). All these differing results drove us to establish a hypothesis about the clinical results that characterize a G12D pattern, never studied before in a differential way. For that reason it is necessary to stratify patients in the different stages according to the (American Joint Committee on Cancer [AJCC], 2009) in order to generate data from a homogeneous population and analyze different mutation patterns. In this line, many groups have investigated the association between *KRAS* mutations and the prognosis of patients in a defined stage of disease (Imamura et al., 2012). Lee et al. (2015) could demonstrate an association between *KRAS* and the prognosis of patients in a homogeneous study cohort of stage III patients treated with FOLFOX. We conducted our study in the same research line.

In the present homogeneous cohort of stage IV Argentinean patients treated with the FOLFOX regimen, we could observe *KRAS* mutations associated with a high-risk of recurrence of disease. Specially, G12D mutations showed a significantly worse clinical outcome. The detrimental effect of *KRAS* mutations was not shown in the analysis of OS. Only the G12S mutation pattern showed poorer survival than the WT, but the small number of patients with this mutation did not allow strong conclusions to be made. We also found patients with WT *KRAS* status who could have benefitted from receiving anti-EGFR therapy in combination with the traditional regimen who did not receive this therapy. The number of patients in this group was around 50% of the WT. Although each patient is unique and generalize is very difficult, this was probably due to regional socioeconomic factors. As already mentioned, the anti-EGFR therapies are not produced in our country. Argentine import policies of foreign products, along with a fragmented health system where patients are dependent on the approval of therapies by private health services delay the entry of drugs into the country. Many patients can die or progress to very advanced stages of their disease before they have access to therapies. Clearly, this does not allow the optimal treatment of patients by physicians.

Another limitation of our study was losing patients due to inconsistent or missing information when searching or tracking lost patients due to their transfer to other regions of the country. We did not take into account the doses of the drugs used, at time to review the clinical records showed slight variations between doses in different patients. Finally, the sample size was insufficient to test the meaning of infrequent *KRAS* subtypes and, more important, analyze their clinical impact in patients.

Strengths include the use of electronic medical history, as it provides important information on how to avoid bias and apply criteria for inclusion in the patient population studied. A database of this type allows a quick though retrospective study that generates evidence which can be very valuable in bringing studies of cancer patients to the bedside. This is the first study that specifically aims to evaluate the clinical impact of specific mutational patterns contralaterally, taking into account how the treatment modality performed, besides being the first study of its kind conducted on a South American population.

In summary, in this analysis of patients with mCRC treated with the FOLFOX regimen, the *KRAS* mutation was independently associated with poorer PFS. Mutation patterns of *KRAS* had different prognostic implications. Studies involving more patients to validate these findings are expected in the future.

## Conflict of Interest Statement

The authors declare that the research was conducted in the absence of any commercial or financial relationships that could be construed as a potential conflict of interest.
